# Coenzyme Q0 from *Antrodia cinnamomea* in Submerged Cultures Induces Reactive Oxygen Species-Mediated Apoptosis in A549 Human Lung Cancer Cells

**DOI:** 10.1155/2014/246748

**Published:** 2014-11-06

**Authors:** Cheng-Han Chung, Szu-Chien Yeh, Chun-Jen Chen, Kung-Ta Lee

**Affiliations:** ^1^Department of Biochemical Science and Technology, National Taiwan University, No. 1, Roosevelt Road Section 4, Taipei 10617, Taiwan; ^2^The Development Center for Biotechnology, No. 101, Lane 169, Kangning Street, New Taipei City 22180, Taiwan

## Abstract

We investigated the anticancer effects of *Antrodia cinnamomea*, a medicinal mushroom from Taiwan, on A549 human lung cancer cells using the ethyl acetate extract from submerged culture filtrates. Our results showed that 2,3-dimethoxy-5-methyl-1,4-benzoquinone (coenzyme Q0; CoQ0) derived from *A. cinnamomea* submerged culture filtrates has anticancer activity. CoQ0 treatment reduced the viability of A549, HepG2, and SW480 cancer cell lines. Furthermore, CoQ0 induced reactive oxygen species (ROS) generation and apoptosis in A549 cells, which was inhibited by the antioxidant ascorbic acid. To our knowledge, these data demonstrate for the first time that CoQ0 derived from *A. cinnamomea* submerged culture filtrates exerts its anticancer effect through the induction of ROS-mediated apoptosis in A549 human lung cancer cells.

## 1. Introduction

*Antrodia cinnamomea *is a parasitic fungus found in Taiwan on the inner cavity of the endemic species* Cinnamomum kanehirae* [1]. This fungus has been used as a traditional medicine for the prevention or treatment of various diseases, including liver disease, food intoxication, drug intoxication, hypertension, and cancer [2]. The fruiting body of* A. cinnamomea* is expensive and rarely found in nature because of its slow growth rate and the limited availability of* C. kanehirae* trees. In addition to fruiting body cultivation,* Antrodia cinnamomea* can be cultured using solid-state or submerged mycelia cultures [2]. The use of submerged cultures to produce pharmacologically active substances from cultured mycelia or broth is beneficial to overcome the slow growth rate of fruiting bodies [3–5]. Thus, isolation and characterization of bioactive compounds from submerged cultures are important for the practical use of* A. cinnamomea* in alternative medicine.

A number of studies have used* A. cinnamomea *submerged cultures to produce bioactive compounds with various pharmacological functions, including antioxidants [6], immunomodulatory [7], hepatoprotective [8], and anticancer agents [9–11]. Several reports indicate that components of the methanolic and ethanolic extracts of* A. cinnamomea* mycelia can inhibit the growth of cancer cells [9, 11]. Antroquinonol is a novel compound isolated from* A. cinnamomea* that displays anticancer activity in human hepatocellular carcinoma, pancreatic carcinoma, and non-small-cell lung carcinoma cells [12, 13]. It has received Food and Drug Administration Investigational New Drug (FDA IND) approval and is undergoing human clinical trials as a new drug in Taiwan and the United States. 4-acetyl antroquinonol B was also isolated from* A. cinnamomea* mycelia and can inhibit the proliferation of HepG2 liver cancer cells [14]. In addition to mycelia, the submerged culture broth of* A. cinnamomea *also exhibits anticancer activity. The filtrate from submerged* A. cinnamomea* cultures inhibits human ovarian carcinoma cell proliferation [15] and induces apoptosis in MCF-7 breast cancer cells* in vitro* and* in vivo* [10, 16]. Previous studies have identified new and known compounds in the submerged culture broth [10, 16–18]; however, the compounds in the culture filtrate that exhibit anticancer activity are still unknown.

In this study, the anticancer effects of the broth filtrate from* A. cinnamomea *submerged cultures were investigated using A549 human lung cancer cells. Lung cancer is the leading cause of cancer-related morbidity in many countries [19]. Thus, there is an urgent need to identify novel, effective treatments. Extracts from various medicinal mushrooms are known to have antitumor properties, making them potential sources for new anticancer compounds [20]. Studies have shown that the submerged culture filtrates from* A. cinnamomea *have antitumor activity [10, 21]; however, the compounds responsible for this effect remain unknown. We aimed to identify the anticancer components in submerged culture filtrates from* A. cinnamomea*. Our results showed that 2,3-dimethoxy-5-methyl-1,4-benzoquinone (coenzyme Q0; CoQ0) was the major anticancer compound in submerged* A. cinnamomea* culture filtrates and that CoQ0 induced apoptosis in A549 cells by stimulating the generation of reactive oxygen species (ROS). Furthermore, we monitored the level of CoQ0 production during the fermentation process.

## 2. Materials and Methods

### 2.1. Reagents and Chemicals

CoQ0 standard and ascorbic acid were purchased from Sigma-Aldrich (St. Louis, MO, USA). Malt extract and peptone were purchased from BD Bacto (Sparks, MD, USA). All other chemicals were purchased from commercial sources at the highest purity available.

### 2.2. Organism and Inoculums

*A. cinnamomea* (BCRC 35716) was obtained from the Bioresource Collection and Research Center (BCRC) at the Food Industry Research and Development Institute (Hsinchu, Taiwan). The mycelium culture was maintained on petri dishes (medium components: 20 g/L glucose, 20 g/L malt extract, 1 g/L peptone, and 20 g/L agar).

### 2.3. Submerged Fermentation

A 4-week mycelium culture on agar was inoculated into 100 mL of preculture medium (20 g/L glucose, 20 g/L sucrose, 2 g/L peptone, pH 5.0) in a 500-mL flask and incubated for 7 days at 25°C on a rotary shaker (100 rpm). The mycelia were then inoculated in a 5-L fermenter (B. Braun Biotech International, Melsungen, Germany) with 3 L of medium. The medium consisted of 20 g/L glucose, 20 g/L malt extract, 2 g/L peptone, 0.3 g/L MgSO_4_, 0.3 g/L KH_2_PO_4_, and 0.3 g/L K_2_HPO_4._ The pH of the medium was adjusted to 5. After inoculation with 1% inoculants, fermentation was carried out at 28°C, 1 vvm, and 100 rpm for 28 days.

### 2.4. Preparation of Broth Extracts

The fermentation product was harvested every week and was separated using filter papers (ADVANTEC, Tokyo, Japan) into culture filtrates and mycelia. The culture filtrate was extracted with an equal volume of ethyl acetate and then vacuum dried. For the cell assay, the extract was dissolved in dimethyl sulfoxide (Sigma-Aldrich) and passed through a 0.2 mm filter. The* A. cinnamomea* submerged culture filtrate extract (ACFE) was stored at −20°C until use in experiments.

### 2.5. Cell Culture

Human A549 lung cancer cells, HepG2 hepatoma cells, and SW480 colon cancer cells were purchased from the Bioresource Collection & Research Centre (Food Industry Research and Development Institute, Hsinchu, Taiwan). The cells were cultured in Dulbecco's Modified Eagle Medium (DMEM) supplemented with 10% FBS (v/v), penicillin (100 units/mL), and streptomycin (100 mg/mL). Cells were cultured in a humidified incubator at 37°C with 5% CO_2_ in air.

### 2.6. Activity Guided Purification of Anticancer Compounds

Thin layer chromatography (TLC) plates were prepared by making slurry of 30 mg of silica gel G (Merck, Darmstadt, Germany) with 60 mL of distilled water. Spreading was done manually over glass plates (20 × 20 cm) and air dried. The plates were activated in an oven for 3 h at 110°C. ACFE was fractionated by petroleum ether (Merck) into two fractions, namely, petroleum ether soluble (PES) and petroleum ether insoluble (PEI). PES (50 mg sample dissolved in 500 *μ*L of ethyl acetate) was used for TLC spotting. Separation of the TLC spots was done using chloroform : ethyl acetate (8 : 2, v/v) as mobile phase. Spots developed on TLC plates were observed under visible light. Spots were eluted by ethyl acetate separately and isolated fractions were tested for their inhibition against A549 cancer cells. The bioactive fraction was then analyzed by the GC-MS and ^1^H NMR.

### 2.7. Cell Viability Analysis

The 3-(4,5-Dimethylthiazol-2-yl)-2,5-iphenyltetrazolium bromide) (MTT) assay was used to determine cell viability. Cells were seeded in 96-well plates (1 × 10^4^ cells/well) overnight in DMEM medium and then treated with different concentrations of ACFE. The effect of CoQ0 on cell growth was examined with the MTT assay. Briefly, 100 *μ*L of MTT solution (2 mg/mL in PBS; Sigma-Aldrich) was added to each well and incubated for 2 h at 37°C. The supernatant was aspirated, and the MTT-formazan crystals formed by metabolically viable cells were dissolved in 100 *μ*L of DMSO. The absorbance was measured by a microplate reader at a wavelength of 570 nm.

### 2.8. GC-MS Analysis

GC-MS analysis was performed using an Agilent GC-MS system (6890N GC system and 5973N mass selective detector, CA, USA) equipped with a HP-5 capillary column (5% diphenyl and 95% dimethyl polysiloxane phase; 0.25 *μ*m film thickness and 30 m × 0.25 mm i.d.). The electron ionization (El) source was operated at 230°C. Helium was used as the carrier gas at a constant flow rate of 0.8 mL/min. The injection volume was 1 *μ*L, and the injection port was maintained at 250°C. The column temperature protocol was 70°C for 2 min with a ramp rate of 3°C/min to 160°C and then 20°C/min to 280°C, where the temperature was maintained for 20 min. The total run time was 58 min. Data were acquired in a full scan mode with *m*/*z* range 50–550 using ChemStation software (Hewlett-Packard, Waldbronn, Germany). Computer searches using the NIST Ver. 2.1 MS data library were employed to identify the spectrum of compounds found by the GC-MS results.

### 2.9. ^1^H NMR Measurements at 400 MHz

NMR measurements were performed on a Bruker Avance 400 Ultrashield spectrometer (Bruker Biospin, Rheinstetten, Germany) equipped with a 5-mm PABBO BB-probe head, using a Bruker Automatic Sample Changer (B-ACS 120). ^1^H NMR spectra were acquired at 300.0 K, and number of scans was 64. The sample was dissolved in CDCl_3_, and tetramethylsilane was used as the internal standard. Chemical shifts were expressed in ppm.

### 2.10. Apoptosis Analysis

Early apoptotic cells were detected using the Alexa Fluor 488 Annexin-V/propidium iodide (PI) kit (Invitrogen, Carlsbad, CA). The apoptosis of A549 cells (1 × 10^6^ cells/well) was measured 24 h after, and cells treated with PBS containing 0.1% DMSO served as negative control. The experimental groups were treated with various concentrations of CoQ0. The quantification of PI and FITC signals was analyzed by a FACSCanto II flow cytometer (BD Bioscience, San Diego, CA, USA). The percentage of stained cells in each quadrant was analyzed using FlowJo software (FlowJo, LLC, Ashland, OR, USA). Cells positive for Annexin-V FITC and negative for PI (Q4 quadrant) were in early apoptosis, and cells positive for both Annexin-V FITC and PI (Q2 quadrant) were in late apoptosis or necrosis.

### 2.11. Determination of the Intracellular ROS Level

To evaluate the intracellular ROS level in A549 cells, the cells were incubated with CoQ0 (25 *μ*g/mL) for the indicated periods. Cells were then incubated with 10 *μ*M 2′,7′-dichlorofluorescin diacetate (DCF-DA) for 30 min prior to harvesting. The presence of ROS causes DCF-DA to be oxidized to the fluorescent compound 2′,7′-dichlorofluorescein (DCF). The fluorescence intensity of the cells was analyzed by flow cytometry.

### 2.12. Statistical Analysis

The results are presented as the mean ± standard deviation (mean ± SD). All study data were analyzed using analysis of variance followed by Dunnett's test for pair-wise comparison. An asterisk indicates that the experimental values were significantly different from those of the controls (^*^ *P* < 0.05).

## 3. Results

### 3.1. Ethyl Acetate Extract from* A. cinnamomea* Submerged Culture Filtrates Inhibits Cancer Cell Survival

**Figure 1:**
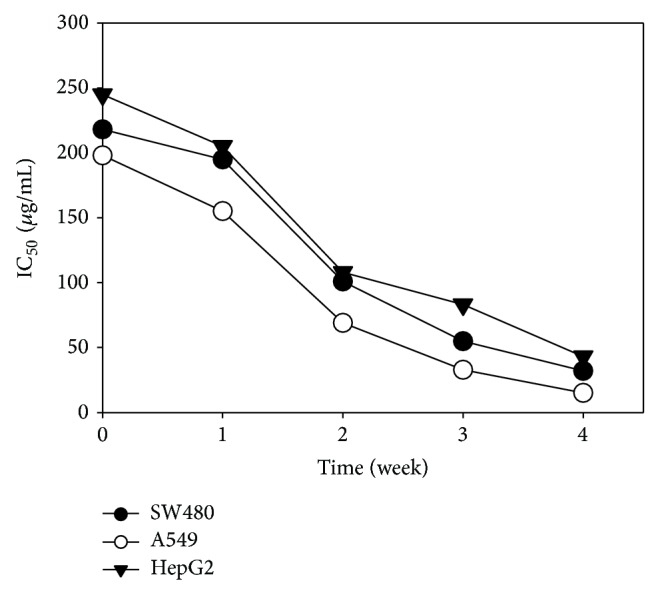
The IC_50_ of ACFE was prepared at different culture times on SW480, A549, and HepG2. The IC_50 _values were measured by the MTT assay as described in the Section 2.

To investigate the anticancer activity of the ACFE and identify the bioactive compound, we first examined the effect of ACFE on the survival of three cancer cell lines, HepG2, A549, and SW480. Cells were treated with ACFE prepared at different culture times, and cell viability was determined by the MTT assay. The results showed that ACFE inhibited the viability of all three cancer cell lines, and the inhibitory effect was stronger when ACFE was prepared at later culture times (Figure 1). In the following experiments, we used the ACFE from 4-week culture filtrates to identify the compound(s) with anticancer activities.

### 3.2. Isolation and Identification of CoQ0 as the Major Anticancer Compound in* A. cinnamomea* Submerged Culture Extract

**Figure 2:**
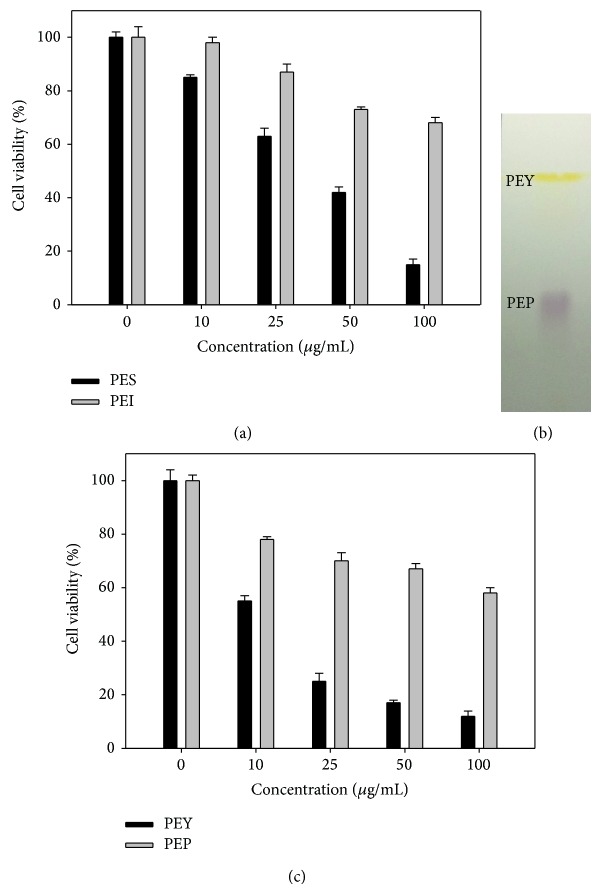
Isolation of anticancer compounds from ACFE prepared at 4-week cultures. (a) Effects of PES and PEI fractions on the viability of A549 cells were measured by the MTT assay. (b) PES fraction was separated by TLC using silica gel G-coated plates and chloroform : ethyl acetate (8 : 2) as mobile phase. (c) Effects of PEP and PEY fractions on the viability of A549 cells were measured by the MTT assay. Data shown are representative of three independent experiments with similar results.

**Figure 3:**
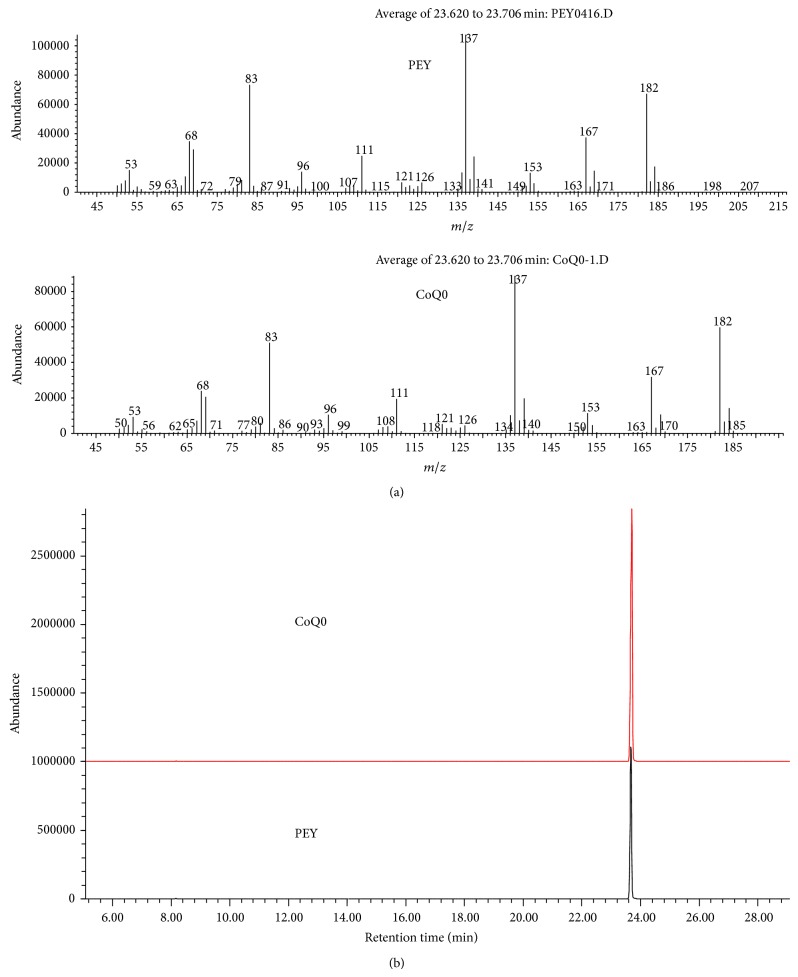
Identification of CoQ0 in PEY by GC-MS. (a) PEY and the CoQ0 standard were analyzed by GC-MS. (b) Mass spectra (MS-EI) of PEY and the CoQ0 standard.

To purify the anticancer compound(s) in ACFE, we first fractionated ACFE by petrol ester into two fractions, namely, the soluble PES and the insoluble PEI fractions, which were then evaluated for their anticancer effects using A549 cancer cells. We found that the PES fraction was more potent than the PEI fraction in suppressing A549 cell proliferation (Figure 2(a)). Components in the PES fraction were further separated by TLC, and the chromatographic profile showed two major bands on the TLC plate under visible light: a purple band (PEP) and a yellow band (PEY) (Figure 2(b)). The PEP and PEY fractions were tested for their anticancer activity by the MTT assay (Figure 2(c)), and the results indicated that the major anticancer activity resided in the PEY fraction. The identity of PEY was first analyzed by GC-MS analysis. The GC-MS spectrum of PEY was identical to that of 2,3-dimethoxy-5-methyl-p-benzoquinone (CoQ0) in the fragment library (Figure 3(a)). PEY and the CoQ0 standard also had the same spectra in the GC-MS total ion chromatograms (TICs) (Figure 3(b)). The PEY: ^1^H NMR (CDCl_3_) d 2.04 (d, *J* = 1.6 Hz, 3H), 3.99 (s, 3H), 4.02 (s, 3H), 6.43 (q, *J* = 1.6 Hz, 1H). The data were similar to CoQ0 reported in literature [22] and the authentic compound. The purification method yielded 65 mg of PE and 16 mg of PEY from 300 mg of ACFE, and the purity of CoQ0 in PEY was 95.5%. The results identified that the major anticancer compound isolated from the submerged culture filtrate of* A. cinnamomea* was CoQ0.

### 3.3. CoQ0 Induced Apoptosis of A549 Lung Cancer Cells

**Figure 4:**
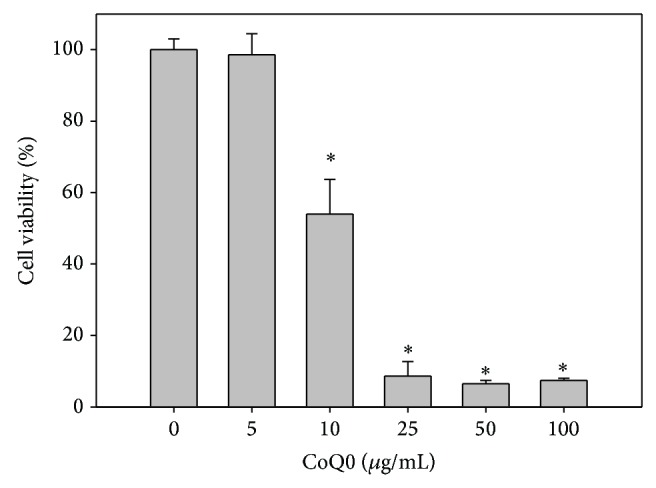
Effect of CoQ0 on the viability of A549 cells. A549 cells were treated with various concentrations of CoQ0 (0 to 100 *μ*g/mL), and cell viability was measured by the MTT assay. Data shown are representative of three independent experiments with similar results. ^*^ *P* < 0.05 versus the control group.

**Figure 5:**
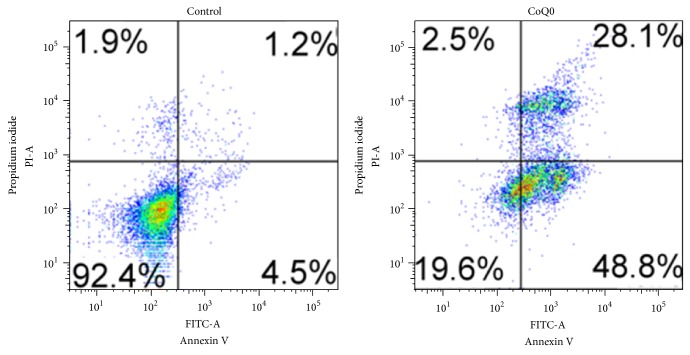
CoQ0 induces apoptosis in A549 cells. A549 cells were treated with CoQ0 (15 *μ*g/mL) for 6 h, and apoptosis was measured by Annexin V/PI staining and flow cytometry. Data shown are representative of three independent experiments with similar results.

To confirm that CoQ0 is a cytotoxic agent for the A549 lung cancer cell, we examined the effect of CoQ0 treatment on cell viability by the MTT assay. CoQ0 showed does-dependent inhibition on A549 cell viability, with an IC_50_ value of approximately 15 *μ*g/mL (Figure 4). We then examined the induction of apoptosis in A549 cells using the Annexin-V/PI assay. The results showed that CoQ0 treatment resulted in a significant increase in both early apoptotic (Annexin-V^+^PI^−^) and late apoptotic (Annexin-V^+^PI^+^) cells (Figure 5). These results demonstrate that CoQ0 exerts it cytotoxic effect on A549 cells via the induction of apoptotic cell death.

### 3.4. CoQ0 Triggered A549 Cell Apoptosis via Inducing ROS Generation

**Figure 6:**
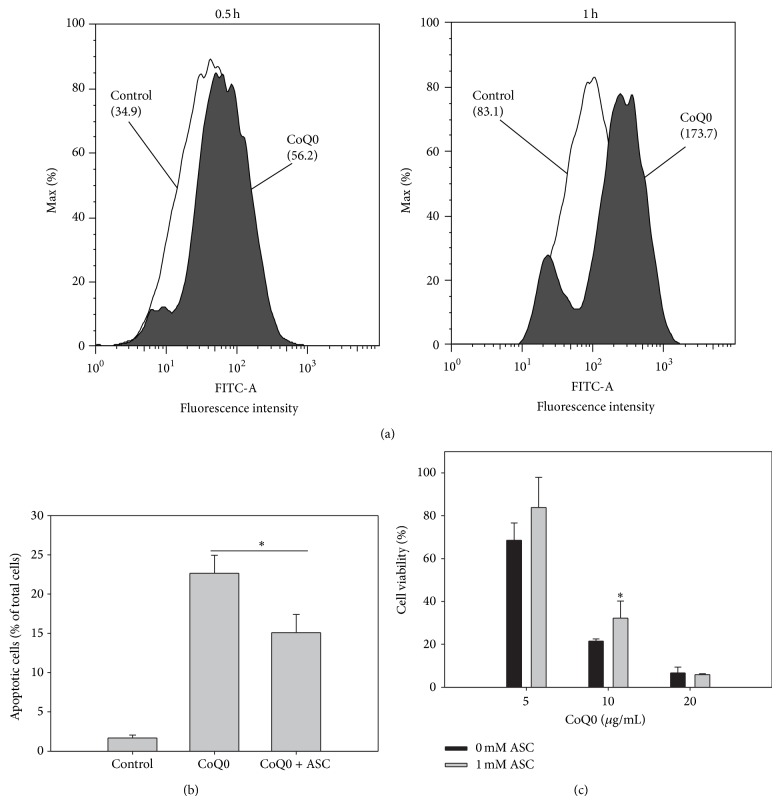
CoQ0 provokes ROS-mediated apoptosis. (a) A549 cells were treated with 15 *μ*g/mL of CoQ0 for 0.5 and 1 h, and the ROS levels were measured by DCF-DA staining and flow cytometry. Numbers in parentheses indicate mean fluorescence intensities. (b) A549 cells were pretreated without or with ASC (1 mM) for 1 h and then incubated with CoQ0 (15 *μ*g/mL) for 6 h. Apoptotic cells were measured by the Annexin V/PI assay. (c) A549 cells were pretreated without or with ASC (1 mM) for 1 h and then incubated with different concentrations of CoQ0 for 24 h. Cell viability was measured by the MTT assay. Data shown are representative of three independent experiments with similar results. ^*^ *P* < 0.05.

ROS play an important role in the induction of apoptosis [23]. To determine if CoQ0 induced apoptosis of A549 cells through ROS accumulation, we used the DCF-DA fluorescent dye to detect the levels of superoxide radicals and hydrogen peroxide in the cell. The results showed that CoQ0 induced a rapid production of intracellular ROS at 0.5 h after treatment (Figure 6(a)). To study if ROS generation was directly associated with CoQ0-induced apoptosis, we assessed apoptotic events in A549 cells pretreated with the antioxidant ascorbic acid (ASC) for 1 h before CoQ0 treatment. ASC is a thiol-free antioxidant and should not interact directly with CoQ0 [24]. Pretreatment with ASC effectively blocked CoQ0-induced apoptosis (Figure 6(b)) and loss of cell viability (Figure 6(c)) of A549 cells. These data clearly demonstrate that CoQ0-induced production of ROS plays an essential role in trigging apoptosis of A549 cells.

### 3.5. Production of CoQ0 by* A. cinnamomea* Submerged Culture in a 5-L Fermenter

**Figure 7:**
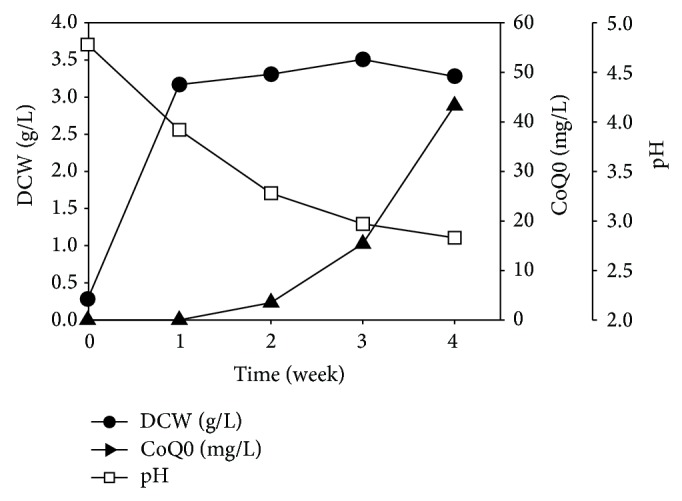
Time course of mycelial growth and CoQ0 production during submerged culture of* A. cinnamomea *in a 5-L fermentor. DCW: dry cell weight.

After identifying CoQ0 as the key anticancer compound in* A. cinnamomea* culture filtrates and characterizing its anticancer mechanism, we next performed a scaled-up mycelial culture of* A. cinnamomea* in a 5-L fermenter and monitored the kinetics of CoQ0 production. As shown in Figure 7, the mycelial biomass reached the highest level of 3.51 g/L on day 21, and CoQ0 appeared after 2 weeks of culture. The concentration of CoQ0 in culture filtrates increased from weeks 2 to 4, and the maximum CoQ0 content of 43 mg/L was obtained after 4 weeks of culture. These results indicate that accumulation of CoQ0 occurs later than biomass formation in* A. cinnamomea* submerged culture.

## 4. Discussion

Fruiting body of* A. cinnamomea* in the wild is well-known as an effective and expensive folk remedy for cancer, in Taiwan, and most studies on the anticancer function of* A. cinnamomea* have examined extracts from the fruiting bodies or mycelia. In this study, we demonstrate that the ethyl acetate extract of the culture broth of* A. cinnamomea* has anticancer activity and identify that CoQ0 is the major cytotoxic compound produced by* A. cinnamomea* during the submerged mycelial culture. Using human A549 lung cancer cell line as a model to characterize the mechanism of CoQ0-induced cancer cell death, our results showed that CoQ0 treatment results in the generation of ROS, which leads to the induction of apoptosis of A549 cells. Batch culture of* A. cinnamomea* in a 5-L fermenter indicated that the maximum level of CoQ0 was produced at a later stage of fermentation. To our knowledge, this is the first report to show that CoQ0 is an anticancer compound produced by the submerged culture of* A. cinnamomea*.

Our data showed that ACFE is cytotoxic to multiple cancer cell lines, including HepG2 hepatoma cells, A549 lung cancer cells, and SW480 colon cancer cells. Through a series of purification steps, we have identified that CoQ0 is the major cytotoxic compound in ACFE. CoQ0 was also shown to modulate cell cycle and induce apoptosis in estrogen receptor-negative human breast cancer cells [25]. These data together suggest that CoQ0 has a general cytotoxicity against various cancer cells and can potentially be developed into an anticancer drug. Similar to our finding, CoQ0 and other quinones have been isolated from the culture broth of* A. cinnamomea* [26] and the mycelium of* Antrodia salmonea *[27]. Many compounds with anticancer activities have been isolated from the fruiting body of* A. cinnamomea*, such as antcin A, methyl antcinate A, and antroquinonol [2, 24]. Interestingly, CoQ0 has not been identified as a cytotoxic compound from the extract of* A. cinnamomea* fruiting body, indicating that different culture methods (fruiting body versus submerged mycelial culture) can result in the production of different metabolites with anticancer activities. These metabolites may kill cancer cells through different mechanisms and may be used for treating different types of cancers. Since the culture broth of* A. cinnamomea* does not contain the anticancer compounds that have been isolated from the fruiting body and/or mycelia, submerged culture media may not replace fruiting body and/or solid-state mycelial cultures for the production of anticancer metabolites other than CoQ0. Studies are now underway to isolate anticancer compounds from the mycelia in submerged culture, and it is likely that mycelia and culture filtrates of* A. cinnamomea* can be used for the production of different anticancer compounds.

Quinones are a class of highly reactive compounds, and they are known to induce cytotoxicity through two major mechanisms: generation of ROS via redox cycling and arylation/alkylation of intracellular nucleophiles such as cysteinyl thiols [28, 29]. Previous studies have suggested that the anticancer mechanisms of certain quinones may involve the production of ROS [30]. ROS are a family of active molecules containing free radicals, and excessive ROS levels cause oxidative stress that may induce cancer cell apoptosis [31]. Our results showed that CoQ0 treatment can increase ROS generation in A549 cells. Furthermore, CoQ0-induced A549 cell apoptosis is attenuated following treatment with the antioxidant ASC (Figure 6(b)). These results demonstrate that ROS play a pivotal role in CoQ0-induced cell death in A549 cells. How does CoQ0 treatment induce ROS-mediated apoptosis? CoQ0 may induce the opening of mitochondrial permeability transition pore (PTP) and the production of H_2_O_2_ as previously described [32, 33]. Similar to our findings, ROS was also shown to mediate apoptosis of HER-2/neu-overexpressing breast cancer cells induced by the culture broth of* A. cinnamomea* [34]. We found that CoQ0 exerts a higher cytotoxicity in A549 cancer cells (IC_50_ ≈ 15 *μ*g/mL) than in normal human fibroblast Detroit 551 cells (IC_50_ ≈ 42 *μ*g/mL, data not shown). In a previous study, CoQ0 was shown to inhibit PTP opening in primary rat hepatocytes and cultured rat liver Clone-9 cells, whereas it induced PTP opening in cancerous rat liver MH1C1 cells [33]. These data suggest that CoQ0 may induce stronger ROS production and cell death in cancer cells than in normal cells. However, since CoQ0 was shown to be toxic to neuron [35]. and insulin-producing cells [33, 36], the dose of CoQ0 that gives selective toxicity to cancer cells should be examined in more detail before it can be considered for use in cancer chemotherapy.

In the current study, we investigated the kinetics of mycelial growth and CoQ0 production of* A. cinnamomea* in a 5-L fermenter. The production of CoQ0 was evident after 2 weeks of submerged culture, when the mycelial growth had reached the stationary phase (Figure 7). The level of CoQ0 in the culture filtrate continued to rise till week 4, the endpoint of culture in this study. The time course of CoQ0 accumulation correlated well with the cytotoxicity of ACFE prepared at different culture times (Figure 1), consistent with our finding that CoQ0 is the major anticancer compound in the culture broth of* A. cinnamomea*. We predict that the amount of CoQ0 might accumulate further when the culture time is extended. Our data suggest that submerged culture of* A. cinnamomea* can be a new way of CoQ0 production besides chemical synthesis [22]. One advantage of producing CoQ0 using the submerged culture of* A. cinnamomea* is that additional anticancer compounds can also be isolated from the mycelia [9, 11].

## 5. Conclusions

*A. cinnamomea* is a medicinal mushroom found in Taiwan and has been used for the prevention or treatment of various diseases [2]. Our results provide evidence that CoQ0 in the filtrate from submerged* A. cinnamomea* cultures induces ROS-mediated apoptosis in A549 human lung cancer cells. Future studies will focus on the anticancer activity and safety profile of CoQ0* in vivo*, which will determine whether CoQ0 has a potential to be developed into a novel chemotherapeutic agent for cancer treatment.
